# Characteristics of the PI3K/AKT and MAPK/ERK pathways involved in the maintenance of self-renewal in lung cancer stem-like cells

**DOI:** 10.7150/ijbs.57871

**Published:** 2021-03-15

**Authors:** Jingyuan Li, Jianyu Wang, Dan Xie, Qin Pei, Xue Wan, H.Rosie Xing, Ting Ye

**Affiliations:** 1 Department of Laboratory Medicine, the Affiliated Hospital of Southwest Medical University, Sichuan, China.; 2 Institute of Life Sciences, Chongqing Medical University, Chongqing, China.; 3 College of Biomedical Engineering, State Key Laboratory of Ultrasound Engineering in Medicine, Chongqing Medical University, Chongqing, China.

**Keywords:** lung cancer stem-like cells, PI3K/AKT pathway, MAPK/ERK pathway, characteristics, self-renewal.

## Abstract

Lung cancer is the leading cause of cancer-related mortality worldwide due to its early asymptomatic and late metastasis. While cancer stem cells (CSCs) may play a vital role in oncogenesis and development of lung cancer, mechanisms underlying CSCs self‐renewal remain less clear. In the present study, we constructed a clinically relevant CSCs enrichment recognition model and evaluated the potential functions of phosphatidylinositol 3-kinase (PI3K)/AKT pathway (PI3K/AKT) and mitogen-activated protein kinases/extracellular signal-regulated kinase (MAPK/ERK) pathways in lung cancer via bioinformatic analysis, providing the basis for in depth mechanistic inquisition. Experimentally, we confirmed that PI3K/AKT pathway predominantly promotes proliferation through anti-apoptosis in lung adenocarcinoma cells, while MAPK/ERK pathway has an overwhelming superiority in regulating the proliferation in lung CSCs. Further, utilizing stemness score model, LLC-Symmetric Division (LLC-SD) cells and mouse orthotopic lung transplantation model, we elucidated an intricate cross-talk between the oncogenic pathway and the stem cell reprograming pathway that impact stem cell characteristics as well as cancer biology features of lung CSCs both *in vitro* and *in vivo*. In summary, our findings uncovered a new insight that PI3K/AKT and MAPK/ERK pathways as oncogenic signaling pathway and/or stem cell signaling pathway act distinctively and synergistically to regulate lung CSCs self-renewal.

## 1. Introduction

Lung cancer is the leading cause of cancer-related mortality worldwide 1. Thereinto, lung adenocarcinoma (LUAD) is one of the most common subtypes of lung cancer based on histologic features 2. Despite advances in diagnostics, surgical techniques as well as new chemotherapy and radiotherapy protocols, the survival rate of lung adenocarcinoma remains only at 4-17% 3.

The hypothesis that cancer stem cells cause tumorigenesis has opened a new frontier in cancer research 4. CSCs possess extensive self-renewal abilities and multi-potent differentiation potentials 5, which promote malignant proliferation, invasion, drug resistance, recurrence and metastasis of various tumors, even with lung adenocarcinoma 6, 7. While CSCs may play a vital role in oncogenesis and development, mechanisms underlying CSCs self‐renewal and the relationship between self‐renewal of the normal stem cells and CSCs remain less clear. Moreover, the drawbacks of current methods of CSCs screening and obtaining are the low yield, unstable generations and the high heterogeneity 8, 9, which prohibited in depth mechanistic investigation. To overcome this technical obstacle, we have developed the stable mouse LLC-SD cell lines established from our previous study for the biological and mechanistic investigation in our present study 10, 11.

The wide application of high-throughput molecular detection technology has caused an explosive increase in molecular omics data 12. And the two largest public databases of tumor gene expression profiles Gene Expression Omnibus 13 (GEO) and The Cancer Genome Atla projects 14 (TCGA) provide a momentous foundation for large-scale tumor analysis research. Thus, in this present study, we constructed a cancer stem-like cell content assessment model built on stem-like characteristic gene enrichment landscape and transcriptome data of lung CSCs. Of note, the stemness scores model achieved the efficiency of estimating the stem-like cell content of tumor tissue based on lung adenocarcinoma transcriptome data, which further verified to be closely associated with the stemness of lung CSCs in evaluating the diagnosis and prognosis of lung adenocarcinoma.

The self-renewal of lung CSCs is closely related to the abnormal activation or inactivation of signal transduction pathways, multiple signal transduction cascades activate the stemness and tumorigenicity of lung adenocarcinoma stem cells 15-19. Hereinto, PI3K/AKT as one of the most commonly oncogenic signaling pathways, promotes tumor growth 20, 21 and enhances stemness via epithelial-to-mesenchymal transition (EMT) in CSCs 22. And yet, recent research indicated that PI3K/AKT pathway can induce progenitor cells differentiation and inhibit renewal 23. In addition, MAPK/ERK pathway has been shown to play an important role in tumor cells growth and differentiation 24, as well as exhibiting cardinal oncogenic roles in lung adenocarcinoma 25, 26. Nevertheless, a recent set of published study has put forward that the MAPK/ERK pathway motivates the stemness features of lung cancer cells by regulating GLI1 27. In general, the characteristics and biological functions of PI3K/AKT and MAPK/ERK pathways as oncogenic signaling pathways and/or stem cell signaling pathways in lung CSCs have not been thoroughly elucidated.

In this study, through the bioinformatics data enriched by stemness scores, we uncovered the potential function and regulatory mechanism of PI3K/AKT pathway and MAPK/ERK pathway in lung adenocarcinoma CSCs. Moreover, utilizing LLC-SD cells and syngeneic orthotopic lung cancer model that we established and characterized 28, we have revealed that PI3K/AKT pathway enhanced the tumorigenicity by promoting the stemness of lung CSCs, while MAPK/ERK pathway promoted the proliferation and differentiation of lung CSCs to drive oncogenesis. In summary, our study provides new insights into how the major oncogenic pathways and/or stem cell pathways, such as the PI3K/AKT and MAPK/ERK signaling pathways, act distinctively and synergistically to regulate lung CSCs self-renewal.

## 2. Materials and methods

### 2.1. Construction of stemness score model based on tumor transcriptome profiles

StemChecker 29 (http://stemchecker.sysbiolab.eu/) was used to collect 26 gene sets related to stemness of stem cells. We used the GEO (http://www.ncbi.nlm.nih.gov/geo) database to screen out GSE35603 dataset with lung adenocarcinoma stem cells and lung adenocarcinoma parental cells. The stemness enrichment score in different gene sets of each sample was calculated via single-sample gene set enrichment analysis 30, 31 (ssGSEA) in this GEO dataset. Differentiate analysis was used to evaluate the ability of 26 gene sets to distinguish CSCs and parental cancer cells, and 10 gene sets with strong discriminative ability were screened out (*P* < 0.05). And the transcriptome expression profile data of TCGA-LUAD dataset was obtained from TCGA (https://portal.gdc.cancer.gov/). Furthermore, the consistency of the enrichment scoring patterns of these 10 gene sets in TCGA-LUAD was analyzed. The final stemness assessment model of LUAD from at least 4/10 gene sets was verified to assess the content of stem-like cells in tumor tissues through GSE35603 and GSE54712. And the stemness score of TCGA-LUAD was calculated by this recognition model.

### 2.2. Verification of stemness score model in TCGA database

The LUAD dataset with clinical information and overall survival times was obtained from TCGA. The original data was normalized and analyzed by the edgeR analysis method. Lung adenocarcinoma data with different high stemness score were divided into high group and low group, between which the expression levels of stem markers were confirmed. Then the levels of stemness score were compared between the lung cancer and normal lung tissues, lung cancer and corresponding adjacent tissues, as well as early and advanced lung adenocarcinoma. Furthermore, receiver operator characteristic curve (ROC), the Kaplan-Meier survival and Cox regression analysis were performed to prove diagnostic and prognostic efficacy of stemness score.

### 2.3. Gene set enrichment analysis (GSEA)

GSEA (http://www.broad.mit.edu/gsea) was performed to identify KEGG (Kyoto Encyclopedia of Genes and Genomes) signaling pathways and the biological functions associated with PI3K/AKT and MAPK/ERK pathways in LUAD dataset with stemness score. Lung adenocarcinoma data were grouped according to the expression of key molecules of PI3K/AKT and MAPK/ERK pathways using RNA-seq data. And then lung adenocarcinoma data were divided into high group and low group according to the differential levels of key molecules in pathways. The expression levels of signaling pathways were analyzed with the functional (curated) dataset from the Molecular Signature Database of the Broad Institute (www.broadinstitute.org/gsea) 31.

### 2.4. Cell culture and cell lines

LLC-Parental cells were cultured in Dulbecco's Modified Eagle Medium (DMEM) /high glucose (Hyclone, USA) containing 10% FBS (Gibco, USA). LLC-SD cells grew with sphere-forming and suspending features under the condition of serum-free DMEM/ Nutrient Mixture F-12 (Hyclone, USA) containing 2% B27 Supplement (Gibco, USA), and were generated as we previously reported 28.

### 2.5. Reverse transcription quantitative real-time polymerase chain reaction (RT-qPCR)

Total RNA was isolated using TRIZOL solution (Takara, Janpan) and reverse-transcribed using the reverse transcription PCR system (Eppendorf, Germany). RT-qPCR was performed with TB Green PCR Master (Takara, Janpan) by Bio-rad qPCR system. Combinations of primer sequences and probe numbers were as follows:

Gene: Forward/Reverse primer (5' to 3')/probe

PI3K: GCAGAGGGCTACCAGTACAGA/CTGAATCCAAGTGCCACTAAGG; Akt: ACTCATTCCAGACCCACGAC/CACAATCTCCGCACCATAGA; mTOR: AGACCTTGAGTTGGCTGTGC/CCTCTGCTTGGATGTGATGA; Eif4e: TGTGGGTAGCAGAGTGGAAA/CAACAAGAGCAGGCGGTTAT; Mek1: GTGCAGTCGGACATCTGGAG/CCACATGGCATCCAAACAGT; Mapk1: CTTCCAACCTCCTGCTGAAC/TCTGTCAAGAACCCTGTGTGA; ALDH1A1: ATACTTGTCGGATTTAGGAGGCT/GGGCCTATCTTCCAAATGAAC; CD133: CTCCCATCAGTGGATAGAGAACT/ATACCCCCTTTTGACGAGGCT; NANOG: TTAGAAGCGTGGGTCTTGGT/CCCTCAAACTCCTGGTCCTT; SOX2: AGGGCTGGGAGAAAGAAGAG/ATCTGGCGGAGAATAGTTGG; TBP: AGGGATTCAGGAAGACCACA/ATGCTGCCACCTGTAACTGA.

### 2.6. Western blot (WB)

Cells were harvested in RIPA lysis buffer with 1% PMSF (Beyotime, China), which was centrifuged at 10,000 × g for 5 min at 4 °C, the supernatant was collected. And protein content was measured using BCA protein quantitative kit (Bio-Rad, USA). Protein expression was determined by the western blot as described 10. The following primary antibodies were used: anti-PI3K (Cell Signaling Technology, USA), anti-Akt (Cell Signaling Technology, USA), anti-mTOR (Cell Signaling Technology, USA), anti-S6k (Cell Signaling Technology, USA), anti-Eif4e (Cell Signaling Technology, USA), anti-Erk1/2 (Cell Signaling Technology, USA), anti-Mek1 (upstate, USA) and anti-GAPDH (Proteintech, China).

### 2.7. Cell counting kit-8 (CCK-8) assay

CCK-8 assay was used to detect cell proliferation. Cells treated with inhibitors were inoculated into 96-well plates with 2 × 10^3^ cells per well, and cells treated with Dimethyl Sulfoxide (DMSO) were used as the control group (3 duplicate wells for each group). After every 24 hours, CCK-8 solution (10 µl/well) was added to each well and incubated at 37 ℃ for 2 hours. The optical density (OD) value at 450 nm was determined by microplate reader (Enspire, Singapore).

### 2.8. Flow cytometric analysis

Cell apoptosis was monitored by the Annexin V-FITC/PI Apoptosis Detection Kit (BD, USA). Cells were added to the 6-well plate and grown for 24 hours before transfection. After 48 hours, cells were washed twice in Phosphate Buffered Saline (PBS, Hyclone, USA) before resuspension in 500 μl PBS Buffer. Next, cells were mixed with annexin V-FITC and propidium iodide (PI) at 37°C for 30 mins. Stained cells were measured by flow cytometry. The data were analyzed via BD FACS software.

### 2.9. Construction of plasmid vectors and stable transfection of lentiviral vectors expression short hairpin RNA (shRNA)

Lentivirus containing shRNA against p110 or Mek1/2 was constructed as we previously described 10. LLC-SD cells were conventionally cultured in medium without antibiotics. At the density of approximately 70%-80%, 1 × 10^6^ virus titers were added and mixed with Eagle's Minimum Essential Medium (MEM) (Hyclone, USA) including 1%FBS. After 12 hours, the completed medium was added to the cells and the previous medium was removed. After 72 hours, cells were then subcultured into the 6-well plates.

### 2.10. Soft agar colony formation assay

Two hundred cells were suspended in two milliliter medium with 0.2% agar, and were poured in six-well plate 32. After 2 weeks, colonies were stained by 0.05% crystal violet. The number of colonies was counted and analyzed.

### 2.11. Single cell colony formation assay

Cells under logarithmic growth phase were diluted into gradient concentrations, then a single cell was seeded into 96-well plate and cultured in an incubator for 2 weeks until the cell colonies were visible (>50 cells were considered a colony) 33. The colonies were observed and counted with microscope.

### 2.12. Animals

Female BALB/c nude mice were purchased from the Beijing HFK Bio-Technology CO., LTD (Beijing, China) and C57BL/6 mice were purchased from the animal center of Chongqing Medical University. The mice were 6-8 weeks old and weighed 18-20g. The study was performed in accordance with Chongqing Management Approach of Laboratory Animal for work with laboratory animals and was approved by The Ethics Committee of Chongqing Medical University in accordance with institutional guidelines.

### 2.13. Nude mice subcutaneous tumors

Cultured cell suspension was mixed with Matrigel Matrix (Corning, USA) at 1:1 ratio, and 0.1 ml mixture (1×10^4^ cells) was injected subcutaneously into the skin of bilateral hind legs of nude mice. Tumor volumes were estimated using the formula V= 0.5 × (length × width^2^). The mice were killed after the tumor sizes reached about 1000mm^3^.

### 2.14. Orthotopic xenograft animal models

Cell suspension was mixed with Matrigel Matrix, and 25 μl of cell suspension (5×10^5^ cells) was injected into the orthotopic left lung of C57BL/6. Two weeks after the treatment, the mice were killed, then the orthotopic and metastatic lung nodules were evaluated by hematoxylin-eosin (HE) staining analysis.

### 2.15. Statistical Analysis

Data was analyzed by the Student's t-tests with correction for multiple comparisons by the Mann-Whiney method. *P* values < 0.05 were considered to be the significant. Results are presented as means ± SEM. *P < 0.05; ** P < 0.01; *** P < 0.001. For the series of RNA-seq analysis, multiple comparisons were corrected using the Benjamini Hochberg method. *P* values < 0.05 as well as false discovery rate (FDR) values < 0.05 were considered to be significant.

## 3. Results

### 3.1. The clinicopathologic and prognostic importance of stemness score in human lung adenocarcinoma

**Figure 1:**
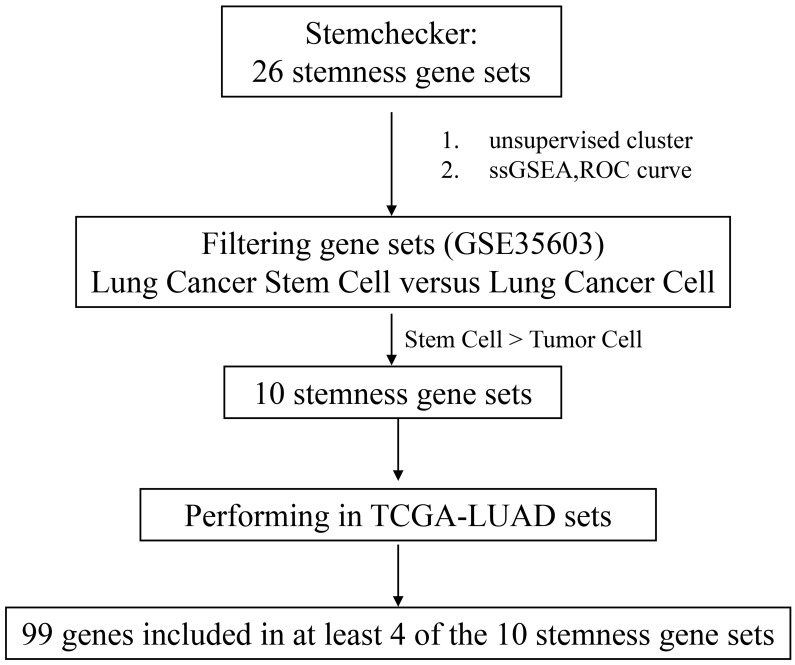
Flow chart of stemness genes screening.

**Figure 2:**
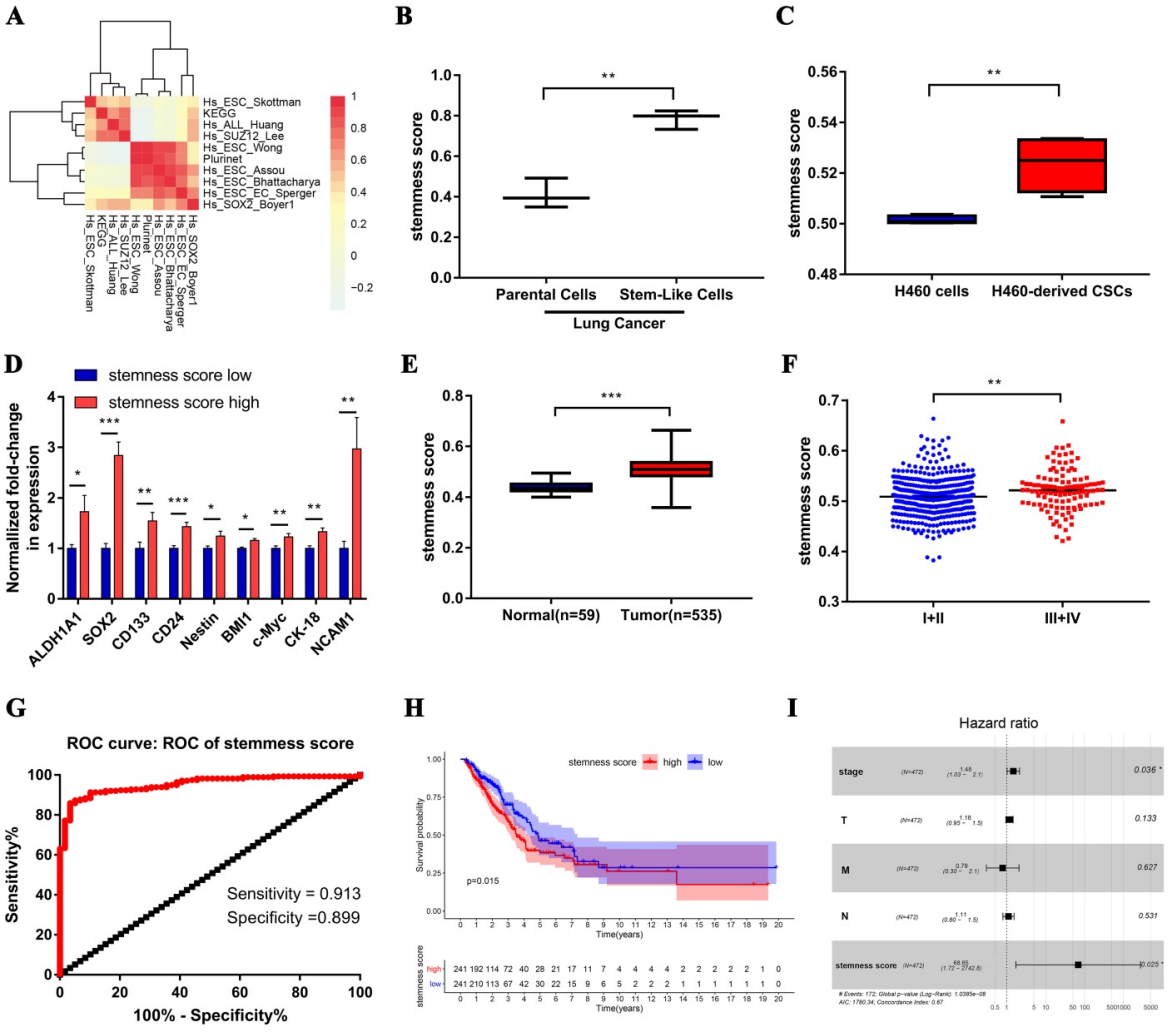
** Stemness score is significantly correlated with clinical characteristics of human lung adenocarcinoma.** A. Correlation heat map of enrichment scores of 10 gene sets in TCGA-LUAD samples. B-C. The chip verified the difference of stemness score between the lung adenocarcinoma cells and lung CSCs, (B) GSE35603, (C) GSE54712. D. The differential expression of stem cell markers in high and low stemness score groups. E. Difference in stemness score between normal (59 samples) and tumor (535 samples) tissues. F. Comparison of stemness score in early and advanced lung adenocarcinoma. G. ROC curve of stemness score in LUAD patients. H. Kaplan-Meier curves showed that the low stemness score group had greater mortality than the high stemness score group. I. Multivariate analysis of the correlation of stemness score with OS among lung adenocarcinoma patients.

We firstly integrated 26 stem-like gene signatures through meta-analysis in Stemchecker database which included previously published stemness gene sets. By taking advantage of these signatures and ssGSEA method, we then obtained the enrichment score of 10 stem-like gene signatures which was dramatically higher in the cancer stem-like cells than that in the cancer parental cells, the specific flow chart was shown in **Figure 1**. Intriguingly, the respective enrichment score of these 10 gene sets showed a noticeable correlativity (**Figure 2A**). Further, the genes existing in more than 4 gene sets, including 99 genes in total, were selected and defined as the stemness score. To evaluate the conspicuous discrimination of stemness score model between the lung adenocarcinoma cells and lung CSCs, we testified this signature which allowed the characterization of positive correlation with stemness through lung CSCs-related expression profiling chips (**Figure 2B-C**). Further, we divided the LUAD cases from TCGA database into high and low groups based on their stemness score and confirmed the expression of stem markers to verify the validation of stemness score model (**Figure 2D**). The results revealed that the expression of stem cell markers (ALDH1A1, SOX2, CD133, CD24, Nestine, BMI1, c-Myc, CK-18, NCAM1) were significantly increased in the high stemness score group compared to that in the low group.

Subsequently, we evaluated the clinical relevance of stemness score in human lung adenocarcinoma. The stemness score was distinctly higher in lung adenocarcinoma than that in normal lung tissue (**Figure 2E**), and was pronounced increase in advanced tumor (**Figure 2F**). Furthermore, the area under the ROC curve (AUC^ROC^) of the stemness score for the early diagnosis of LUAD was 0.953, with the sensitivity of 91.3% and the specificity of 89.9% respectively (**Figure 2G**). Also, survival analysis showed that the high stemness score confers poor prognosis in LUAD patients for overall survival (OS) (**Figure 2H**). In addition, Multivariate Cox regression analysis further suggested that the high stemness score was an independent risk factor affecting the prognosis of LUAD patients (**Figure 2I**, HR = 68.65, *P* = 0.025).

### 3.2. Assessment of the enrichment correlations between the stemness score levels and signaling pathways functions in human lung adenocarcinoma

**Figure 3:**
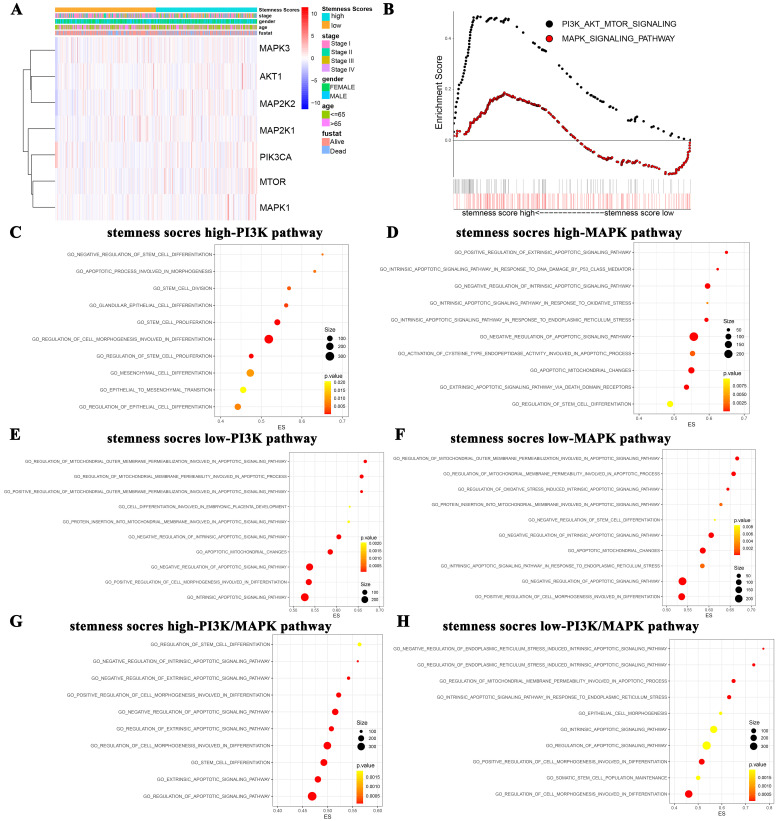
** The function enrichment of PI3K/AKT and MAPK/ERK signaling pathways in the groups with different stemness scores levels.** A. Heat map of correlation between stemness score and signaling molecules at transcriptional level. B. The GSEA showed the PI3K/AKT and MAPK signaling pathway enrichment results using TCGA-LUAD datasets. C-H. Biological process analysis of malignant characteristics of tumors in different groups. (C-D) Enriched results with correlation of PI3K/AKT/mTOR pathway in stemness scores high group (C) and low group (D), respectively. (E-F) Enriched results with correlation of MAPK/ERK pathway in stemness scores high group (E) and low group (F), respectively. (G-H) Enriched results with correlation of PI3K/AKT/mTOR combined MAPK/ERK pathway in stemness scores high group (G) and low group (H), respectively.

We firstly evaluated the enrichment relationship between the stemness score and key molecules of PI3K/AKT pathway involving PIK3CA, AKT1, mTOR and MAPK/ERK pathway involving MAP2K1, MAP2K2, MAPK1 and MAPK3 (**Figure 3A-B**). The KEGG enrichment analysis revealed that PI3K/AKT pathway was markedly enriched in high stemness score group (**Figure 3B**, *P* < 0.05). Moreover, we conducted GO analysis to uncover the most valuable 10 clusters of enriched sets closely associated with proliferation, differentiation, apoptosis, as well as stemness and carcinogenicity characteristics.

Notably, in the high stemness score group, the PI3K/AKT was significantly enriched in the biological process categories of negative regulation of stem cell differentiation, apoptotic process involved in morphogenesis, stem cell division, etc. (**Figure 3C**). And the MAPK/ERK pathway was enriched in positive regulation of extrinsic apoptotic signaling pathway, intrinsic apoptotic signaling pathway in response to DNA damage by p53 class mediator, negative regulation of intrinsic apoptotic signaling pathway, etc. (**Figure 3D**). Whereas, in the low stemness score group, PI3K/AKT pathway was enriched in regulation of mitochondrial outer membrane permeabilization involved in apoptotic signaling pathway, regulation of mitochondrial membrane permeability involved in apoptotic process, positive regulation of mitochondrial outer membrane permeabilization involved in apoptotic signaling pathway, etc. (**Figure 3E**). MAPK/ERK pathway was enriched in regulation of mitochondrial outer membrane permeabilization involved in apoptotic signaling pathway, regulation of mitochondrial membrane permeability involved in apoptotic process, regulation of oxidative stress induced intrinsic apoptotic signaling pathway, etc. (**Figure 3F**). As for the crosstalk enrichment of these two pathways, PI3K/AKT pathway combined with MAPK/ERK pathway in high stemness score group were enriched in regulation of stem cell differentiation, negative regulation of intrinsic apoptotic signaling pathway, negative regulation of extrinsic apoptotic signaling pathway, etc. (**Figure 3G**). PI3K/AKT pathway combined with MAPK/ERK pathway in low stemness score group were enriched in negative regulation of endoplasmic reticulum stress induced intrinsic apoptotic signaling pathway, regulation of endoplasmic reticulum stress induced intrinsic apoptotic signaling pathway, regulation of mitochondrial membrane permeability involved in apoptotic process, etc. (**Figure 3H**). Taken together, GO analysis collectively revealed the close associations between the stemness scores levels and the biological functions of oncogenic and/or stem cell pathways, such as the PI3K/AKT and MAPK/ERK pathways.

### 3.3. The differential characteristics of PI3K/AKT and MAPK/ERK signaling pathways in mediating proliferation between the LLC-P and LLC-SD cells *in vitro*

**Figure 4:**
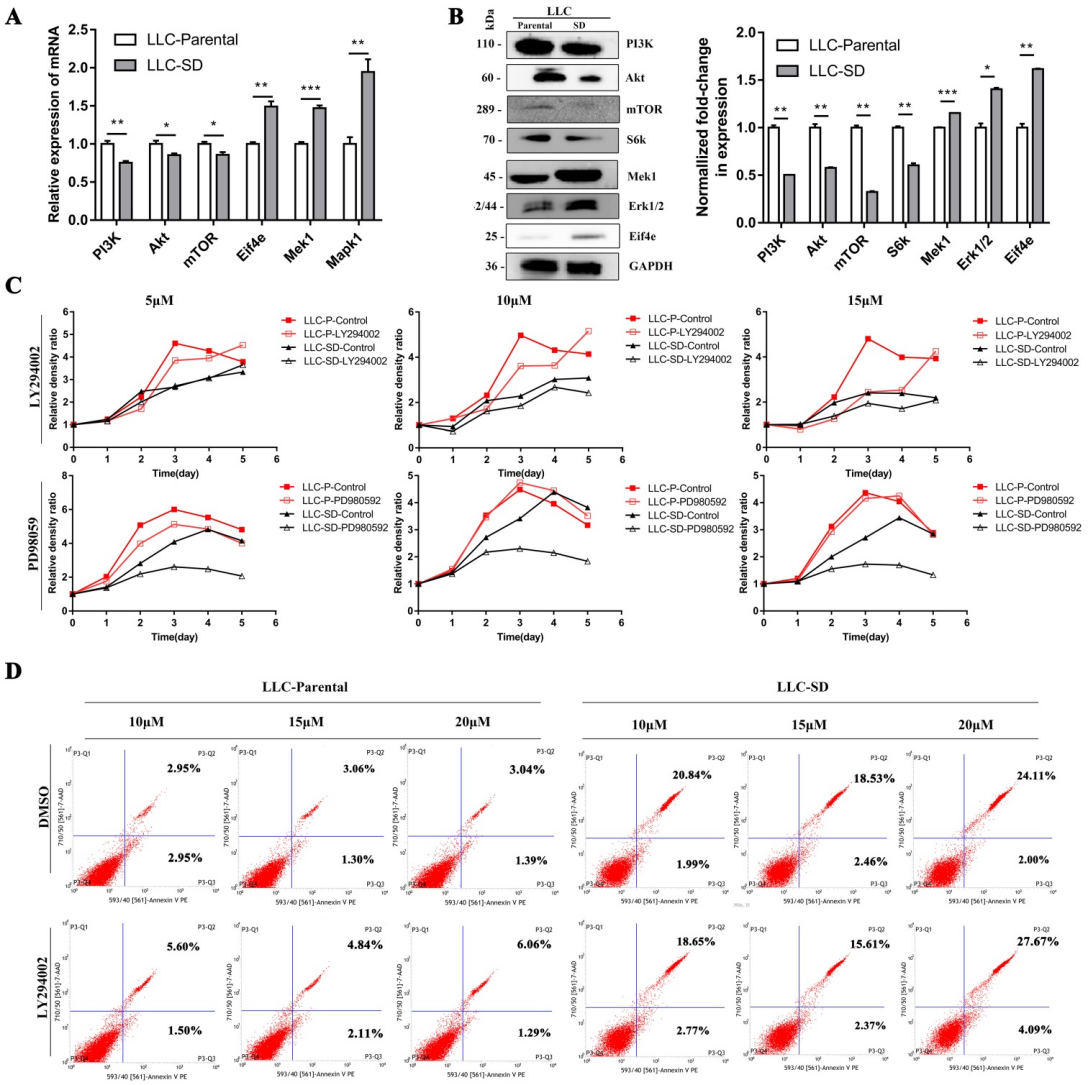
** The differential characteristics of PI3K/AKT and MAPK/ERK signaling pathways in mediating proliferation between the LLC-P and LLC-SD cells *in vitro*.** A-B. Signaling molecules expression in LLC-Parental cells compared with LLC-SD cells as examined by RT‐qPCR (A) and western blot (B). C. LLC-Parental and LLC-SD cells were seeded at 2000/well and treated with increasing concentrations of LY294002 and PD98059, respectively. Cell growth curves were derived and compared to the DMSO control. D. Annexin V-FITC/PI staining of apoptotic LLC-Parental and LLC-SD cells under treatment of LY294002.

The mRNA and protein expression of key signaling components of PI3K/AKT and MAPK/ERK signaling pathways were firstly detected by RT-qPCR and WB in LLC-Parental and LLC-SD cells which differs in stemness levels. The LLC-Parental cells had elevated molecule expressions of PI3K/AKT signaling than the LLC-SD cells, while the MAPK/ERK signaling components were robustly upregulated in LLC-SD (**Figure 4A-B**). To investigate whether PI3K/AKT and/or MAPK/ERK activation are required for the proliferation of LLC-Parental and LLC-SD cells, we suppressed PI3K/AKT pathway with LY294002 and MAPK/ERK pathway with PD98059 inhibitors, respectively. Thereafter, the effect of PI3K/AKT and MAPK/ERK pathway on proliferation was measured by CCK-8 assay. As shown in **Figure 4C**, the proliferative activity of LLC-SD cells was remarkably inhibited upon PD98059 treatment, while LLC-Parental cells were mainly hindered after LY294002 treatment, and even with concentration-dependent manner. Further, we confirmed the mechanism underlying PI3K/AKT facilitation of proliferation in LLC-Parental cells is the anti-apoptosis via flow cytometry analysis (**Figure 4D**).

### 3.4. The PI3K/AKT and MAPK/ERK signaling pathways had opposite effect on the regulation of LLC-SD cells self-renewal* in vitro*

**Figure 5:**
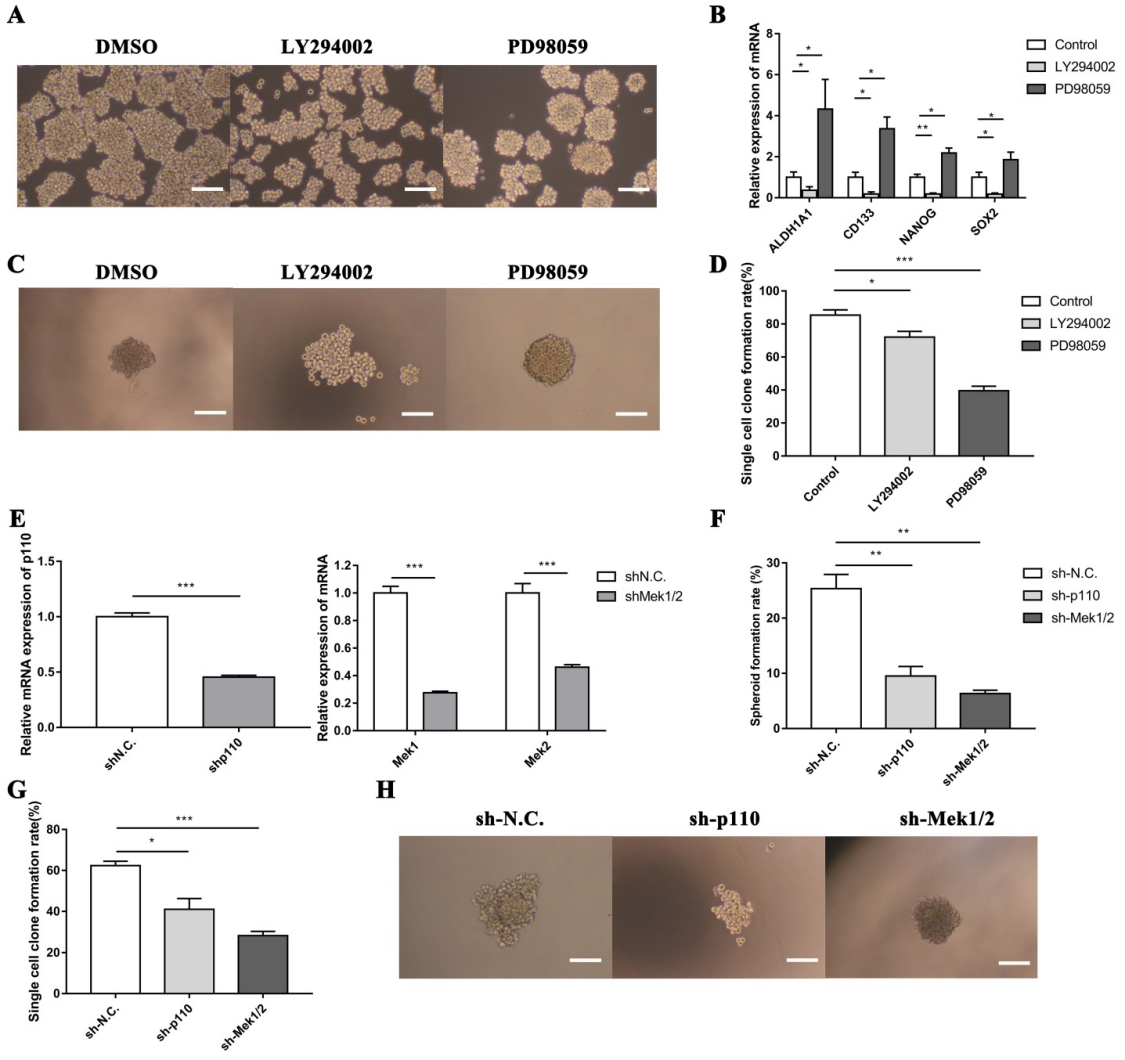
** The PI3K/AKT and MAPK/ERK signaling pathway mediates self-renewal of LLC-SD cells* in vitro*.** A. Cellular morphology of spheroid formation in LLC-SD cells treated with negative control DMSO and with specific inhibitors LY294002 and PD98059. B. RT‐qPCR analysis of mRNA expression of signaling molecules. C. Cellular morphology of single-cell cloning formation in LLC-SD cells treated with LY294002 and PD98059. D. Quantitative analysis of single‐cell cloning formation rate. E. Expression of Mek1, Mek2 and p110 in LLC-SD cells infected with shRNA to Mek1/2 and p110 by RT-qPCR. F-G. Analysis of spheroid formation rate (F) and single‐cell cloning formation rate (G) from shp110 and shMek1/2 cells. H. Morphological differences of single-cell cloning formation in shp110 and shMek1/2 cells. Scale bars, 120 μm.

To confirm the function of PI3K/AKT and MAPK/ERK signaling pathways on stem-like cell properties of LLC-SD cells, we assessed the capability of sphere formation of LLC-SD cells treated with LY294002 or PD98059 by serial mammosphere formation and single cell clone formation analysis. Strikingly, LLC-SD cells exhibited abnormal morphology with incompact structure after inactivation of PI3K/AKT pathway by LY294002. On the contrary, the inhibitory effect of PD98059 on MAPK/ERK pathway enhanced the feature of stem-like spheroid morphology in LLC-SD cells (**Figure 5A**). Furthermore, we identified the expression of stem cell genes after inhibition of PI3K/AKT and MAPK/ERK pathways. As presented in **Figure 5B**, the expression of ALDH1A1, CD133, NANOG and SOX2 were significantly downregulated in LLC-SD treated with LY294002, while these genes expressions were markedly elevated in LLC-SD dealt with PD98059. In agreement with the serial mammosphere formation analysis, the results of single cell clone formation analysis demonstrated that LLC-SD cells displayed loose morphology after LY294002 treatment, whereas the morphology became regular tight stem-like spheroid after PD98059 treatment (**Figure 5C**). But both of the clones rate in LLC-SD cells reduced after inhibitors treatment (**Figure 5D**).

In order to further investigate the involvement of PI3K/AKT and MAPK/ERK pathways in mediating LLC-SD self-renewal, LLC-SD cells were virally transduced with shp110 and shMEK1/2 (**Figure 5E**). The effect of p110 or Mek1/2 silencing on feature of stem-like cells was observed by soft agar colony formation assay and single cell clone formation analysis. These observations are consist with the findings as using the inhibitors treatment (**Figure 5F-H**). From the above, our data supports that the activation of the PI3K/AKT pathway and inhibition of the MAPK/ERK pathway promoted the stemness in lung CSCs.

### *3.5.* The different effects of PI3K/AKT and MAPK/ERK signaling pathways on the tumorigenicity and progression of LLC-SD cells *in vivo*

**Figure 6:**
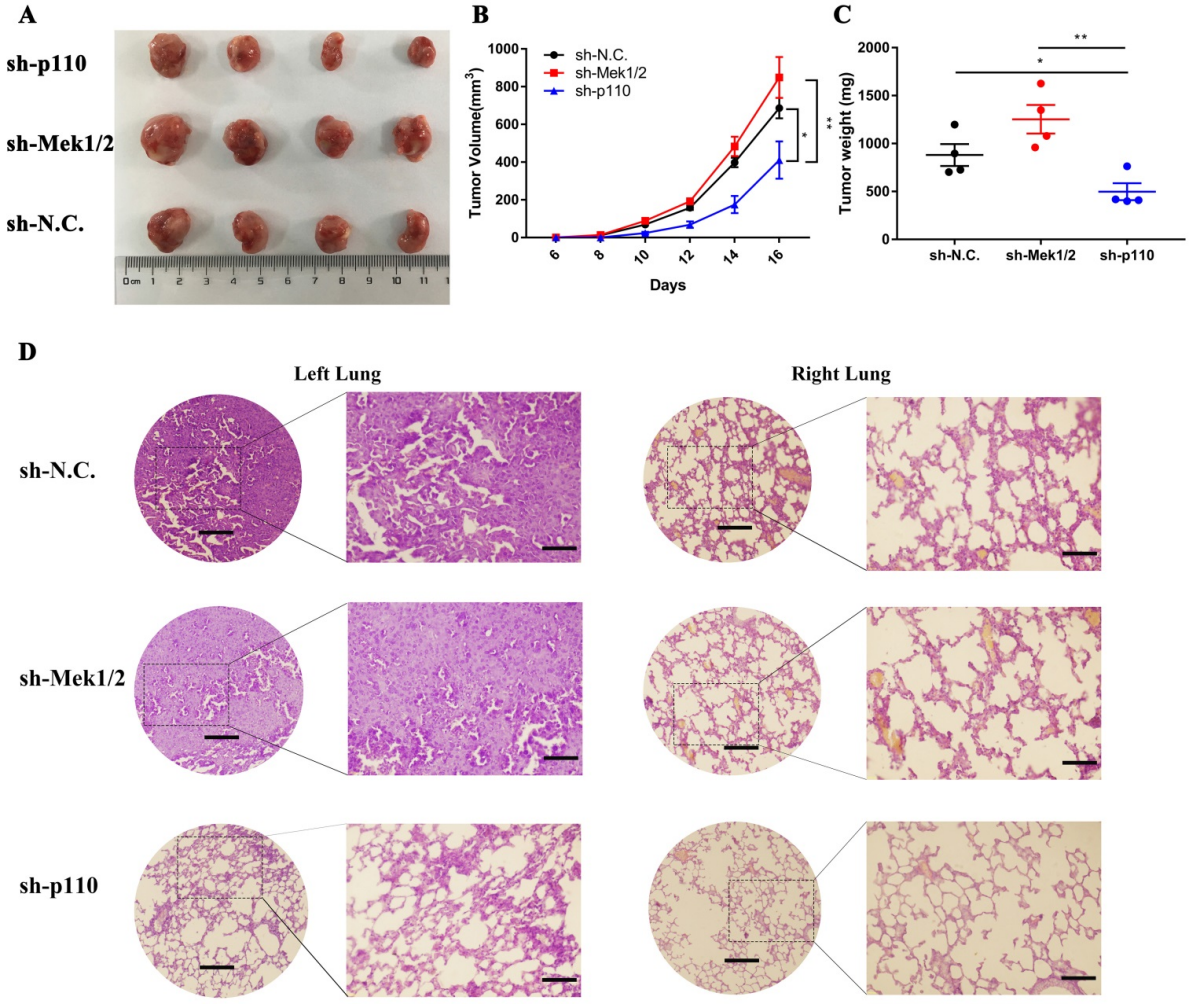
** The PI3K/AKT and MAPK/ERK signaling pathway regulates tumorigenicity and progression of LLC-SD cells *in vivo*.** A-C. Subcutaneous tumors were harvested after LLC-SD infected with shRNA in BALB/c nude mice. (A) tumor volume, (B) tumor growth curve, (C) tumor weight. D. HE staining analysis of orthotopic tumors derived from shp110 and shMek1/2 cells transplanted to the lung of C57BL/6 mice. Scale bars = 120μm, the box indicates the enlarged area, bar = 60μm.

*In vivo*, we first compared the differences in tumorigenic capability between the PI3K/AKT and MAPK/ERK signaling pathways in LLC-SD cells by subcutaneous tumor transplantation assay in BALA/c nude mice. The inactivation of PI3K/AKT pathway by p110 knockdown inhibited the visual size of subcutaneous xenotransplanted tumors (**Figure 6A**), growth kinetics of the tumors (**Figure 6B**) and tumor weight (**Figure 6C**). However, the inhibition of Mek1/2 expression and the resultant inactivation of the MAPK/ERK pathway produced no significant influence in terms of tumor volume and weight compared with LLC-SD-shN.C. control tumors (**Figure 6A-C**). Further, we developed the clinically relevant syngeneic LLC orthotopic mouse model of lung cancer to verify the tumor biology characteristics of LLC-SD after Mek1/2 or p110 knockdown. And the tumors in the left lung (*in situ*) and right lung (metastasis) were removed and analyzed by HE staining. Histology confirmed the more severe presence of the left orthotopic tumor and right lung metastases in mouse received LLC-SD-shN.C. and LLC-SD-shMek1/2 cells (**Figure 6D**). In short, this set of observations are consistent with the *in vitro* evidences, demonstrating the importance of PI3K/AKT and MAPK/ERK pathways on the lung CSCs self-renewal regulation in tumorigenicity and progression *in vivo*.

## 4. Discussion

Accumulating evidence provided more insights into CSCs and the complex effects of different signaling pathways may trigger the switch of tumor cells to a stem cell-like phenotype 34-36. While PI3K/AKT and MAPK/ERK signaling pathways have been reported to be responsible for numerous oncogenic properties of lung adenocarcinoma 37-39, yet the mechanistic characterization of self-renewal maintenance in lung cancer stem-like cells by manipulating oncogenic and stem cell signaling pathways remain limited and elusive. This present study revealed that PI3K/AKT and MAPK/ERK pathways have distinctive and synergistical functions in regulating the fate of lung CSCs, which could represent a new mechanism of cross-talk between the oncogenic and stem cell pathway.

The present study has made the following novel findings that contribute to an improved molecular-mechanistic understanding of self-renewal features in lung CSCs:

First, the clinicopathologic and prognostic importance of stemness score in human lung adenocarcinoma has been uncovered for the first time by bioinformatics analysis. Prior to our study, the evaluation model of the stem-like cell content of tumor tissue based on lung adenocarcinoma transcriptome data has not been explored and established (**Figure 1**). Utilizing the clinically relevant tumor stem cell enrichment recognition model, we obtained the stemness score of tumor samples and normal adjacent to cancer samples in TCGA-LUAD. The high stemness score appears to positively correlate with the stemness level of lung CSCs and exerts staging-wise elevation in lung adenocarcinoma (**Figure 2A-F**). We also found that stemness score plays a pivotal role in evaluating the diagnosis and prognosis of lung adenocarcinoma, thus is of high clinical relevance (**Figure 2G-I**).

Second, based on the bioinformatics data enriched by stemness scores, this study was the first attempt to predict the potential molecular mechanisms underlying the oncogenic and pro-stemness activities of PI3K/AKT and MAPK/ERK signaling pathways in human LUAD. PI3K/AKT and MAPK/ERK signaling pathways have been reported to be the classical oncogenic signaling pathways involved in regulating the tumorigenesis and development 17. Concomitantly, they are also potential stem cell signaling pathways, referring to the regulation of stem cell self-renewal, differentiation and proliferation 40. Hence, we conducted an in-depth discussion on the different functions of two signaling pathways to promote carcinogenesis and regulate self-renewal of lung CSCs via GO analysis (**Figure 3**). As noted previously, in LUAD patients groups with high stemness score, PI3K/AKT signaling pathway is enriched in regulating stem cell proliferation and negatively controlling differentiation, while MAPK/ERK pathway focuses on regulating stem cell differentiation and apoptosis. Interestingly, in low stemness score group, PI3K/AKT and MAPK/ERK signaling pathway both participate in regulating cancerous functions such as apoptosis.

Third, we provide convincing experimental evidence supporting a rather differential role of PI3K/AKT and MAPK/ERK signaling pathways in regulating proliferation of lung cancer cells and CSCs. Using CCK-8 and flow cytometry assays, we conducted that PI3K/AKT signaling pathway predominantly promotes proliferation via resistance to apoptosis in LLC-Parental cells, whereas MAPK/ERK signaling pathway has an overwhelming superiority in regulating proliferation of LLC-SD cells (**Figure 4C and 4D**). Here, these results confirm that PI3K/AKT pathway accelerated proliferation of lung cancer cells and MAPK/ERK pathway facilitated proliferation of lung CSCs, consistent with the different expressions of the PI3K/AKT and MAPK/ERK signaling pathway-related genes between the lung cancer cells and lung CSCs (**Figure 4A and 4B**).

Fourth, we made a preliminary attempt to elucidate an intricate cross-talk between the oncogenic pathway and the stem cell reprogramming pathway that impacts stem cell characteristics as well as cancer biology features of mouse lung CSCs. Specifically, we identified the positive regulation of PI3K/AKT and negative regulation of MAPK/ERK in the maintenance of lung CSC self-renewal and stem cell properties *in vitro* (**Figure 5**). Meanwhile, we didn't stop here and took further observation which uncovered the interplay on promoting LUAD tumourigenesis and development between the PI3K/AKT and MAPK/ERK signaling pathways in lung CSCs through* in vivo* experiments (**Figure 6**). Utilizing the subcutaneous tumor transplantation assay in nude mice and orthotopic lung transplantation models in C57BL/6 mice, we confirmed that inactivation of PI3K/AKT signaling pathway inhibited tumorigenicity and metastasis of lung CSCs, while the inhibition of MAPK/ERK signaling pathway showed no significant changes. Notably, the discrepancy of function experiments upon MAPK/ERK blocking *in vitro* and *in vivo* might be ascribed to the inactivation of MAPK/ERK pathway accompanied by dual inhibition of self-renewal and proliferative activity in LLC-SD cells for tumor expansion. Without doubt, this merits further validation.

Taken together, the study we presented here has provided new insights on the mechanisms in regulating CSCs self-renewal, and elucidated the interplay between the oncogenic pathway and stem cell pathway in lung CSCs that is mediated by PI3K/AKT and MAPK/ERK.

## Abbreviations

CSCs: cancer stem cells
PI3K/AKT: phosphatidylinositol 3-kinase (PI3K)/AKT
MAPK/ERK: mitogen-activated protein kinases/extracellular signal-regulated kinase
LLC-SD: LLC-Symmetric Division
LUAD: lung adenocarcinoma
EMT: epithelial-to-mesenchymal transition
GSEA: gene set enrichment analysis
KEGG: Kyoto Encyclopedia of Genes and Genomes
TCGA: The Cancer Genome Atla projects
GEO: Gene Expression Omnibus
DMEM: Dulbecco's Modified Eagle Medium
DMSO: Dimethyl Sulfoxide
PBS: Phosphate Buffered Saline
OD: optical density
MEM: Eagle's Minimum Essential Medium
HE: hematoxylin-eosin
ROC: Receiver Characteristic Operator
AUC: Area under the ROC curve
